# Defining the Subtypes of Long COVID and Risk Factors for Prolonged Disease: Population-Based Case-Crossover Study

**DOI:** 10.2196/49841

**Published:** 2024-04-30

**Authors:** Skyler Resendez, Steven H Brown, Hugo Sebastian Ruiz Ayala, Prahalad Rangan, Jonathan Nebeker, Diane Montella, Peter L Elkin

**Affiliations:** 1 Department of Biomedical Informatics University at Buffalo, State University of New York Buffalo, NY United States; 2 Office of Health Informatics Department of Veterans Affairs Washington, DC United States

**Keywords:** long COVID, PASC, postacute sequelae of COVID-19, public health, policy initiatives, pandemic, diagnosis, COVID-19 treatment, long COVID cause, health care support, public safety, COVID-19, Veterans Affairs, United States, COVID-19 testing, clinician, mobile phone

## Abstract

**Background:**

There have been over 772 million confirmed cases of COVID-19 worldwide. A significant portion of these infections will lead to long COVID (post–COVID-19 condition) and its attendant morbidities and costs. Numerous life-altering complications have already been associated with the development of long COVID, including chronic fatigue, brain fog, and dangerous heart rhythms.

**Objective:**

We aim to derive an actionable long COVID case definition consisting of significantly increased signs, symptoms, and diagnoses to support pandemic-related clinical, public health, research, and policy initiatives.

**Methods:**

This research employs a case-crossover population-based study using *International Classification of Diseases, 10th Revision, Clinical Modification* (*ICD-10-CM*) data generated at Veterans Affairs medical centers nationwide between January 1, 2020, and August 18, 2022. In total, 367,148 individuals with *ICD-10-CM* data both before and after a positive COVID-19 test were selected for analysis. We compared *ICD-10-CM* codes assigned 1 to 7 months following each patient’s positive test with those assigned up to 6 months prior. Further, 350,315 patients had novel codes assigned during this window of time. We defined signs, symptoms, and diagnoses as being associated with long COVID if they had a novel case frequency of ≥1:1000, and they significantly increased in our entire cohort after a positive test. We present odds ratios with CIs for long COVID signs, symptoms, and diagnoses, organized by *ICD-10-CM* functional groups and medical specialty. We used our definition to assess long COVID risk based on a patient’s demographics, Elixhauser score, vaccination status, and COVID-19 disease severity.

**Results:**

We developed a long COVID definition consisting of 323 *ICD-10-CM* diagnosis codes grouped into 143 *ICD-10-CM* functional groups that were significantly increased in our 367,148 patient post–COVID-19 population. We defined 17 medical-specialty long COVID subtypes such as cardiology long COVID. Patients who were COVID-19–positive developed signs, symptoms, or diagnoses included in our long COVID definition at a proportion of at least 59.7% (268,320/449,450, based on a denominator of all patients who were COVID-19–positive). The long COVID cohort was 8 years older with more comorbidities (2-year Elixhauser score 7.97 in the patients with long COVID vs 4.21 in the patients with non–long COVID). Patients who had a more severe bout of COVID-19, as judged by their minimum oxygen saturation level, were also more likely to develop long COVID.

**Conclusions:**

An actionable, data-driven definition of long COVID can help clinicians screen for and diagnose long COVID, allowing identified patients to be admitted into appropriate monitoring and treatment programs. This long COVID definition can also support public health, research, and policy initiatives. Patients with COVID-19 who are older or have low oxygen saturation levels during their bout of COVID-19, or those who have multiple comorbidities should be preferentially watched for the development of long COVID.

## Introduction

Numerous symptoms are cited as long-term sequelae of COVID-19. “The symptoms may affect a number of organ systems, occur in diverse patterns, and frequently get worse after physical or mental activity” [1]. Early studies found that the most common long-term symptoms were fatigue, dyspnea, joint pain, and chest pain [2]. Others reported gastrointestinal tract disorders correlated with gut microbiome shifts after COVID-19 infection [3,4]. Cognitive dysfunction, often referred to as brain fog, is another commonly reported long-term symptom [5]. Cognitive dysfunction is particularly concerning given evidence that COVID-19 can alter brain structure [6]. The most common self-reported symptoms documented via a smartphone app were fatigue, headache, dyspnea, and anosmia [7,8].

More recent studies have added to the knowledge base concerning symptoms of long COVID (post–COVID-19 condition). For example, a study observing cohorts in 4 Chinese cities showed that fatigue, cough, sore throat, difficulty in concentrating, feeling of anxiety, myalgia, and arthralgia were common severe long COVID symptoms. While there is considerable overlap, there is still value in new studies as they help validate previous studies and add new insights, such as identifying a previously underappreciated increase in anxiety among those who had COVID-19 [9].

Recent reviews have analyzed and integrated long COVID research to date [10]. Reviews like these contribute knowledge (eg, that over 200 symptoms have been identified, affecting multiple organ systems while proposing potential mechanisms). They also point out where our knowledge base is lacking. For example, Davis et al [10] observed a study stating that postural tachycardia syndrome can be a potential complication [11] but research has recently shown that long COVID can also greatly increase the likelihood of complications such as atrial fibrillation [12]. Such contributions are why continued research is critically important to combat the detrimental effects of long COVID.

Concerningly high long COVID frequencies have been reported since near the start of the pandemic. A cohort study from the Netherlands found that approximately 1 in 8 patients with COVID-19 developed long-term somatic symptoms [13]. Another study showed that approximately 30% of their cohort reported persistent symptoms, with many experiencing worse health-related quality of life compared with baseline and negative impacts on at least one activity of daily living [14]. More recent studies confirm these alarming statistics, indicating that 1 in 7 adults in the United States have reported symptoms of long COVID [15]. Furthermore, long COVID’s impacts extend beyond individual morbidity to include the health care system and economic consequences. Cutler [16] noted long COVID resulted in reduced workforce participation (eg, 44% out of the workforce), direct earning losses, and worker shortages in service jobs. This is likely directly related to the fatigue associated with long COVID, which has now been linked to muscular abnormalities and overall dysfunction of mitochondria within these tissues [17].

The widespread occurrence of lingering ailments and their impacts on individuals and society make clear the need for a long COVID definition. US public health officials note that we must balance our need for an accurate long COVID definition that includes all afflicted individuals against our need for interim long COVID definitions to expedite immediate action and mobilization [18]. In particular, a working definition of long COVID based on routinely collected coded data could support the identification of at-risk or undiagnosed patients for monitoring, referral, or therapeutic interventions. In this study, we empirically derive an actionable broad-based long COVID definition to support current clinical, public health, research, and policy initiatives related to the pandemic.

## Methods

### Overview

**Figure 1:**
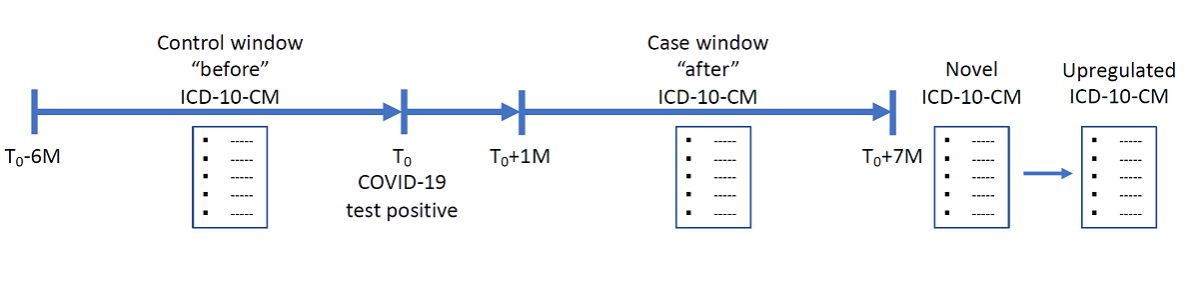
Workflow and data curation in the sequence used to generate our long COVID definition. ICD-10-CM: International Classification of Diseases, 10th Revision, Clinical Modification; M: months; T_0_: date of positive COVID-19 test.

We selected veterans who had laboratory-confirmed positive COVID-19 tests. We examined the veterans’ electronic health records for novel *International Classification of Diseases, 10th Revision, Clinical Modification* (*ICD-10-CM*) codes between 1 and 7 months after a positive COVID-19 test. We grouped codes with a novel frequency of 1/1000 or greater by diagnosis type creating *ICD-10-CM* functional groups and performed *χ*^2^ testing with Bonferroni correction to compare diagnosis frequencies before and after a positive COVID-19 test. We defined *ICD-10-CM* functional groups that significantly increased in frequency as “upregulated” (see Figure 1). We then manually aggregated upregulated *ICD-10-CM* functional groups into medical specialties to organize our empiric definition of long COVID.

### Population Definition and Data Extraction

We selected patients with laboratory-confirmed positive COVID-19 studies and followed them for 13 months (6 months before the COVID-19 test result and 7 months after COVID-19) to create a long COVID definition. We used the electronic health records of all the patients who tested positive for COVID-19 at Veterans Affairs (VA) medical facilities nationally between January 1, 2020, and August 18, 2022. In total, 2,377,720 patients were tested for COVID-19 during this time period.

We applied SQL queries to VA Informatics and Computing Infrastructure, Corporate Data Warehouse data tables [19] to generate 2 diagnosis files for analysis. The first file (“before”) contains a row of retrospectively collected information for each patient and each *ICD-10-CM* diagnosis assigned to them in the 6-month control window before their COVID-19 test. The row includes the *ICD-10-CM* code and its description, a unique patient identifier, the COVID-19 test date, and the calculated number of months between *ICD-10-CM* code entry and COVID-19 testing. This “before” file contained 14,980,288 observations across 426,970 patients.

We followed the patients for 7 months. The second file (“after”) was created 7 months after the last patient was included. The after file contained *ICD-10-CM* codes assigned during the 7 months following COVID-19 testing and similar related information as the “before” file. This “after” file contained 15,493,587 observations across 389,677 patients.

**Figure 2:**
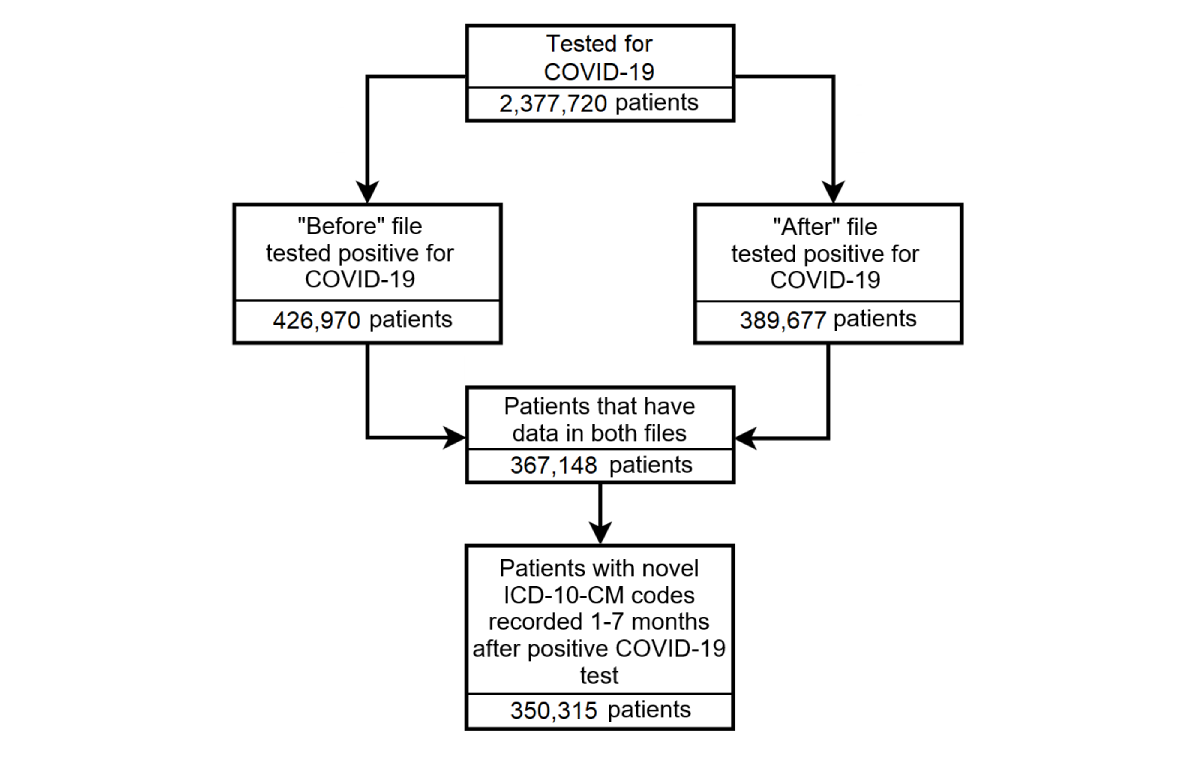
Patient selection flowchart for this study. The first step excludes all patients that test negative for COVID-19. The second step excludes all patients that are not in both the “before” and “after” files, indicating either a lack of diagnosis history or a lack of follow-up, respectively. The final step excludes any patient that does not have a novel diagnosis 1 to 7 months after their positive test. A diagnosis is novel if it was not observed in the “before” file but was observed during this time. The “before” file consists of data concerning every ICD-10-CM code assigned to each patient who was COVID-19 positive in our cohort for up to 6-months before their test. The “after” file consists of data concerning every ICD-10-CM code assigned to each COVID-19 positive patient in our cohort for up to 7-months after their test. ICD-10-CM: International Classification of Diseases, 10th Revision, Clinical Modification.

We limited the analysis to the 367,148 patients that appeared in both the “before” and “after” files to ensure that we had a diagnostic history for each patient and eliminated acute findings by removing all *ICD-10-CM* codes documented less than a month after the positive COVID-19 test (Figure 1). We used the date of the first positive COVID-19 test for patients with multiple positive tests. Multiple repeating *ICD-10-CM* codes for a single patient were counted once. We wrote R (R Development Core Team) and Python (Python Software Foundation) programs to remove all data concerning patients who tested negative for COVID-19, *ICD-10-CM* codes that were documented less than a month after the positive COVID-19 test, and patients who were not present in both the “before” and “after” files. The methodology used to generate the patient cohort is depicted in Figure 2.

We collected additional data to examine the association of demographics, comorbidities, vaccination status, and COVID-19 case severity with the incidence of long COVID. Demographic data collected included age, sex, race, and ethnicity. Comorbidities were evaluated using 2-year Elixhauser Comorbidity Indices Scores. Patients who were vaccinated were defined as having at least one COVID-19 vaccine dose recorded for at least two weeks and no more than 9 months before their positive COVID-19 test. We defined 2 classes of severe COVID-19 based on the minimum recorded oxygen saturation. The first class, severe COVID-19, was defined by a minimum oxygen saturation of <94% [20]. The second class, severe COVID-19 with severe desaturation, was defined by a minimum oxygen saturation of <88% [21].

### Ethical Considerations

An institutional review board protocol was developed and approved for this research by the Department of Veterans Affairs (number 1580090). It uses electronic health care records data for patients of VA hospitals across the country. These data are protected health care data and are only used outside of the VA system in a summarized, deidentified format. The original informed consent from patients allows these analyses without additional consent. No additional compensation was given. A HIPAA (Health Insurance Portability and Accountability Act) waiver was approved (00004461).

### Data Analysis

We chose 6 months “before” and “after” COVID-19 test control and case windows to allow patients to serve as their own controls. The 6 month “after” case window began 1 month after the positive COVID-19 test. We defined “novel” *ICD-10-CM* codes as those that appeared in a patient’s “after” file but not in the “before” file. We calculated the frequency of each novel *ICD-10-CM* code as the percentage of this study’s cohort assigned with the code. We excluded novel codes with a frequency of <1:1000 from further analysis. We defined codes as upregulated when the frequency of that code in the after file was significantly increased as compared to the frequency in the before file, if it had a *χ*^2^ with a Bonferroni corrected *P*<.00006. All resulting codes were grouped for additional analysis and organization (see *ICD-10-CM* Functional and Medical Specialty Groupings subsection of Methods).

We also defined *ICD-10-CM* functional groups as “upregulated” if they were statistically more frequent post COVID-19 by *χ*^2^ analysis with Bonferroni correction with a *P*<.00006. We limited the long COVID ICD-10-CM code functional groups to those that were significantly increased in frequency. We used “before” and “after” frequencies to calculate odds ratios and CIs for each novel ICD-10-CM functional group. We calculated odds ratios from frequency data.

We analyzed potential risk factors including vaccination status and COVID-19 severity for long COVID by creating 2 × 2 tables and applying Pearson *χ*^2^ testing. We applied a similar approach to the analysis of demographic factors. We used R (version 4.1.2) and RStudio (Posit Software, PBC) to perform the statistical analysis.

### ICD-10-CM Functional and Medical Specialty Groupings

We grouped *ICD-10-CM* codes in 3 steps. We first combined *ICD-10-CM* codes that had the same initial 3 characters. We then grouped *ICD-10-CM* codes with different initial characters if the diagnoses were functionally similar to create our *ICD-10-CM* functional groups. For example, we grouped I47.1 (supraventricular tachycardia), I47.2 (ventricular tachycardia), and R00.0 (tachycardia, unspecified) as tachycardia. Finally, we manually curated each of these *ICD-10-CM* functional groups into medical specialties for organizational purposes.

### Long COVID Definition

We included in our long COVID definition each *ICD-10-CM* code with an incidence over 6 months (T_0_ + 1M – T_0_+7M) >1:1000 (M: months; T_0_: date of positive COVID-19 test) and a significant overall frequency increase. Patients with long COVID were defined as having any of the 323 upregulated *ICD-10-CM* codes between 2 and 7 months after their positive COVID-19 diagnosis, but not in their pre–COVID-19 diagnoses.

### Risk Factors for Long COVID

The multivariate regression models were done for each risk factor one including age, gender, race, ethnicity, and 2-year Elixhauser score; a second with age, gender, race, ethnicity, 2-year Elixhauser score, and O_2_ saturation <94%; and a third with age, gender, race, ethnicity, 2-year Elixhauser score, and COVID-19 vaccination status. We present the univariate rates as well as the results of the regression analysis using R (version 4.1.2) and RStudio.

## Results

### Long COVID Definition

We extracted *ICD-10-CM* diagnosis codes assigned to 367,148 patients who underwent a positive COVID-19 test at VA. A total of 268,320 patients had one or more novel COVID-19–related diagnoses. The remaining 98,828 patients had no novel long COVID *ICD-10-CM* diagnoses in their post–COVID-19 period when compared to their pre–COVID-19 period. Table 1 contains the demographic characteristics of this study’s cohort. Men were significantly older than women on average, 60.29 years (95% CI 60.24-60.35) versus 47.85 years (95% CI 47.73-47.97), respectively.

We developed a definition of long COVID consisting of 323 *ICD-10-CM* diagnosis codes grouped into 143 *ICD-10-CM* functional groups that were significantly increased in our 367,148 patient post–COVID-19 population. We define 17 medical specialty long COVID subtypes including cardiology long COVID, neurology long COVID, and pulmonary long COVID. Multimedia Appendix 1 shows the *ICD-10-CM* functional groups and medical specialties. Within each field, the *ICD-10-CM* code groups are sorted in descending order by their odds ratios. Combined odds ratios were calculated for each medical specialty category in Table S2 in Multimedia Appendix 2. Additional information about specific codes can be found in Table S3 in Multimedia Appendix 3.

**Figure 3:**
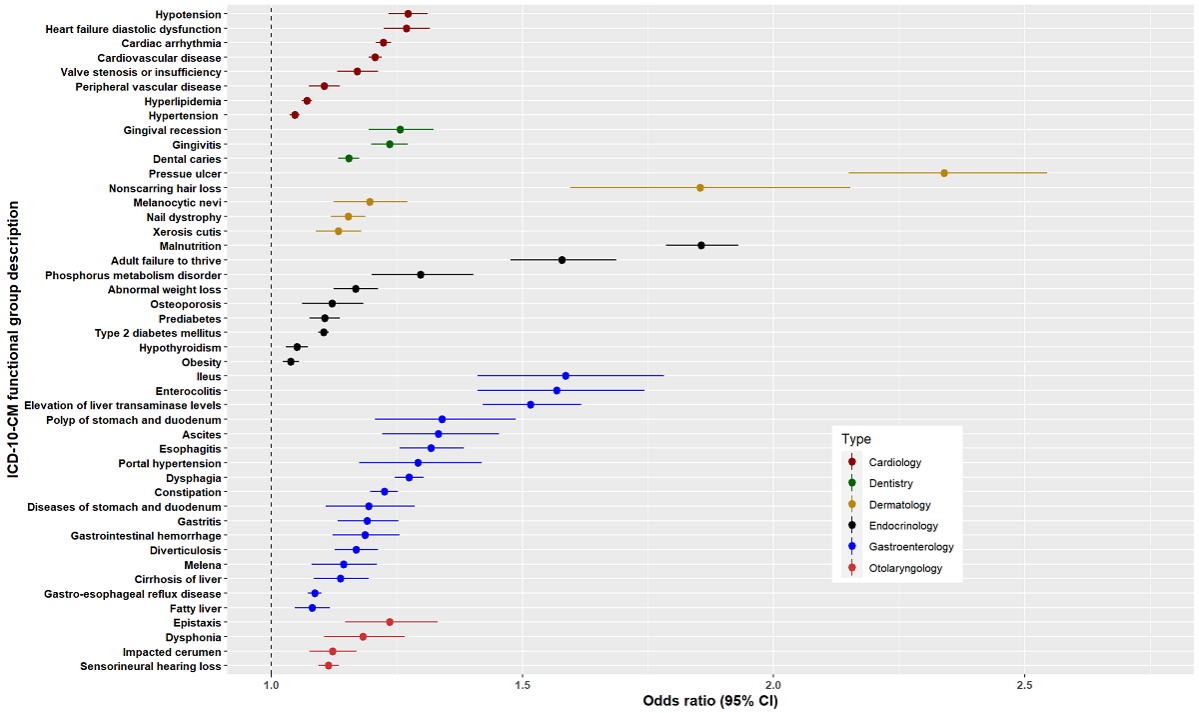
Odds ratios <3 for long COVID ICD-10-CM functional groups by medical specialty subtype: cardiology, dentistry, dermatology, endocrinology, gastroenterology, and otolaryngology. ICD-10-CM: International Classification of Diseases, 10th Revision, Clinical Modification.

**Figure 4:**
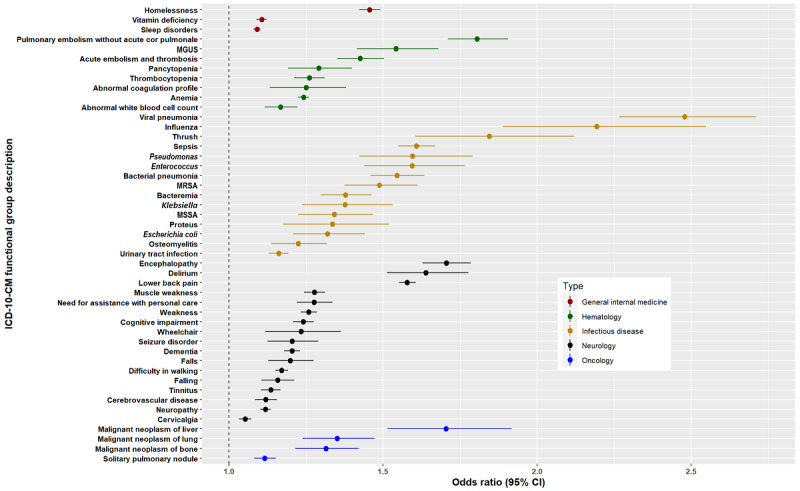
Odds ratios <3 for long COVID ICD-10-CM functional groups by medical specialty subtype: general internal medicine, hematology, infectious disease, neurology, and oncology. ICD-10-CM: International Classification of Diseases, 10th Revision, Clinical Modification. MGUS: monoclonal gammopathy of undetermined significance; MRSA: methicillin-resistant *Staphylococcus aureus*; MSSA: meticillin-sensitive *Staphylococcus aureus*.

**Figure 5:**
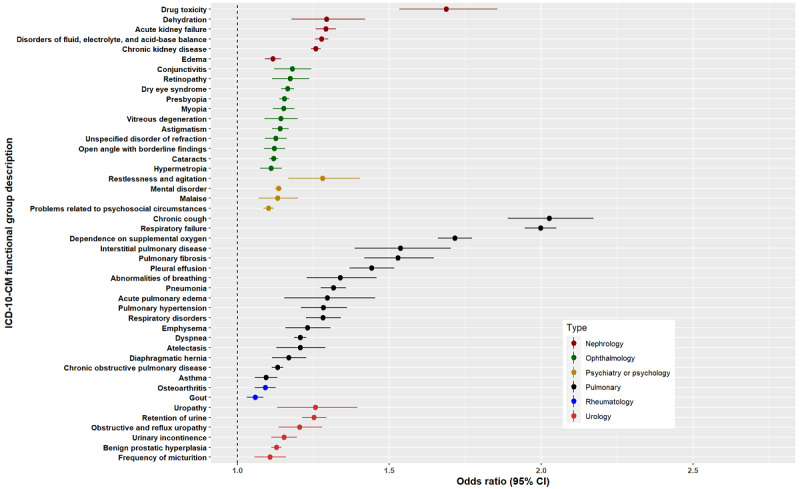
Odds ratios <3 for long COVID ICD-10-CM functional groups by medical specialty subtype: nephrology, ophthalmology, psychiatry or psychology, pulmonary, rheumatology, and urology. ICD-10-CM: International Classification of Diseases, 10th Revision, Clinical Modification.

**Figure 6:**
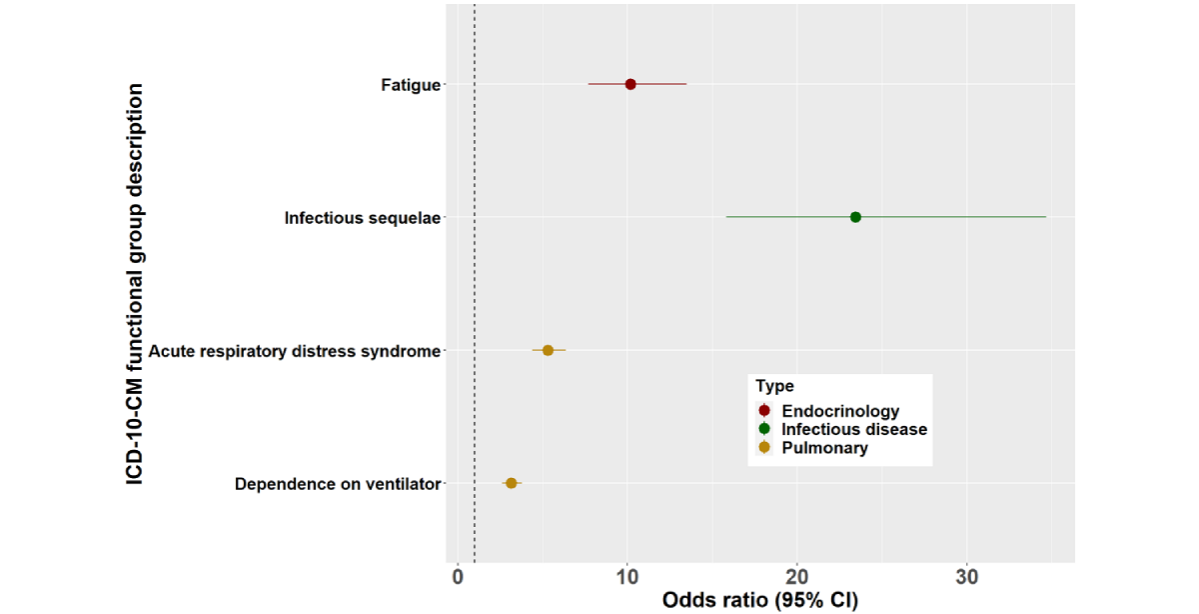
Odds ratios >3 for long COVID ICD-10-CM functional groups by medical specialty subtype. ICD-10-CM: International Classification of Diseases, 10th Revision, Clinical Modification.

Figures 3-6 show the signs, symptoms, and diagnoses with significantly increased relative risks in the post–COVID-19 period with their respective 95% CIs sorted by medical specialty. The data used to create these figures can be found in Table S4 in Multimedia Appendix 4.

Case counts were greatest for the specialties of cardiology (196,632), neurology (159,358), ophthalmology (149,817), and pulmonary (138,470). The lowest case counts were for oncology (7256), rheumatology (10,543), and dermatology (13,233; see Table 2 for more details).

Patients who were COVID-19 positive were assigned novel signs, symptoms, or diagnoses included in our definition of long COVID at a rate of between 59.7% (268,320/449,450, the percentage is based on patients who were COVID-19 positive and tested at the VA) and 76.6% (268,320/350,315, the percentage is based on all patients who were COVID-19 positive with a diagnostic history and follow-up diagnoses 1 to 7 months after test).

Most patients with long COVID were documented with at least one *ICD-10-CM* code found in our long COVID definition within 3 months of their positive COVID-19 test (168,194/268,320, 62.7%). The percentage of patients documented with their first long COVID *ICD-10-CM* code decreased with each subsequent month.

**Table 1:** Demographic data and 2-year Elixhauser scores for patients with long COVID and patients with non–long COVID.

Demographic			Patients with non–long COVID (N=98,828)		Patient with long COVID (N=268,320)		*P* value
Age (years), mean (95% CI)			52.14 (52.03-52.24)		60.85 (60.79-60.91)		—^a^
Elixhauser score, mean (95% CI)			3.03 (2.99-3.07)		7.05 (7.01-7.09)		—
**Gender, n (%)**							
	Men	75,418 (76.31)		234,720 (87.48)		<.001	
	Women	16,854 (17.05)		31,651 (11.8)		<.001	
	Not listed	6556 (6.63)		1949 (0.73)		<.001	
**Ethnicity, n (%)**							
	Hispanic or Latino	10,147 (10.27)		26,171 (9.75)		<.001	
	Not Hispanic or Latino	71,595 (72.44)		227,404 (84.75)		<.001	
	Not listed	17,086 (17.29)		14,745 (5.5)		<.001	
**Race, n (%)**							
	American Indian or Alaska Native	743 (0.75)		2243 (0.84)		.011	
	Asian	1420 (1.44)		2973 (1.11)		<.001	
	Black or African American	20,779 (21.03)		65,218 (24.31)		<.001	
	Native Hawaiian or other Pacific Islander	905 (0.92)		2522 (0.94)		=.50	
	White	55,359 (56.02)		173,169 (64.54)		<.001	
	Not listed	19,622 (19.85)		22,195 (8.27)		<.001	

^a^Not available.

**Table 2:** Case counts by medical specialty.

Subspecialty	Diagnoses	Cases, n
Cardiology	38	196,632
Neurology	38	159,358
Ophthalmology	20	149,817
Pulmonary	42	138,470
Endocrinology	23	97,884
Gastroenterology	27	87,302
Nephrology	33	86,582
Psychiatry or psychology	9	75,292
Hematology	20	46,372
Urology	9	39,336
General internal medicine	9	33,130
Infectious diseases	22	30,998
Dentistry	13	30,202
Otolaryngology	4	23,583
Dermatology	7	13,223
Rheumatology	2	10,543
Oncology	4	7256
Totals	320	1,225,980

### Risk Factors for Long COVID

We presented in Table 1 a comparison of demographic characteristics and Elixhauser comorbidity scores of patients with long COVID and patients with non–long COVID. The long COVID cohort was older with more comorbidities. The long COVID cohort also had higher percentages of White and Black individuals and non-Hispanic and non-Latino ethnicities. The patients with a 2-year Elixhauser score of greater than 21 had a much higher proportion to develop long COVID (*P*<.001, Pearson *χ*^2^; see Table 3).

Our data did not indicate that vaccination was protective against the development of long COVID. However, vaccination resulted in significantly lower rates of novel acute respiratory distress syndrome in the post–COVID-19 period (13.2%, 95% CI 10.4%-16.9%) as compared with the unvaccinated population (19.6%, 95% CI 18.1%-21.2%; *P*<.001).

Patients with minimum O_2_ saturations constituting severe COVID-19 and severe COVID-19 with severe desaturation were significantly more likely to develop long COVID (both had *P*<.001, Pearson *χ*^2^; see Table 4).

The multivariate regression models all confirmed that patients with COVID-19 during the Omicron variant predominant period were at a slightly higher risk of developing long COVID at *P*<.001.

**Table 3:** Proportion of patients that developed long COVID comparing different 2-year Elixhauser score ranges.

2 year Elixhauser score	Non–long COVID count	Long COVID count	Percent long COVID^a^ (95% CI)
0-21	96,055	242,108	71.60 (71.44-71.75)
22-42	2454	22,716	90.25 (89.88-90.61)
43-63	308	3317	91.50 (90.55-92.36)
64-84	11	179	94.21 (89.93-96.77)

^a^The percentages come from the numbers to the left of each percentage. It is the long COVID count divided by the sum of both categories.

**Table 4:** Low oxygen saturations and the proportion of patients that developed long COVID.

Severe COVID-19	Non–long COVID count	Long COVID count	Percent long COVID^a^ (95% CI)
Low O_2_ (NIH^b^ definition^c^)	3903	29,411	88.28 (87.93-88.63)
No low O_2_ (NIH definition^c^)	94,925	238,909	71.57 (71.41-71.72)
Low O_2_ (severe desaturation^d^)	637	5566	89.73 (88.95-90.46)
No low O_2_ (severe desaturation^d^)	98,191	262,754	72.80 (72.65-72.94)

^a^The percentages come from the numbers to the left of each percentage. It is the long COVID count divided by the sum of both categories.

^b^NIH: National Institutes of Health.

^c^Minimum (O_2_ saturation) <94%.

^d^Minimum (O_2_ saturation) <88%.

## Discussion

### Conclusions

Numerous reports document specialty-specific signs, symptoms, and diagnoses correlated with long COVID. We present a novel analysis based on a large national data set and the full multispecialty breadth of *ICD-10-CM* diagnosis codes to create a holistic long COVID definition that confirms and extends previous reports.

We allowed patients to be their own controls and used the entire cohort before and after COVID-19 infection to determine the relative risk of signs, symptoms, and disorders. This ensured that the signal was both novel and upregulated. We found patients who were COVID-19 positive developed signs, symptoms, or diagnoses included in our long COVID definition at a proportion of between 59.7% (268,320/449,450, the percentage is based on a denominator of all patients who were COVID-19 positive and tested at the VA) and 76.6% (268,320/350,315, the percentage is based on a denominator of all patients who were COVID-19 positive with a diagnostic history and follow-up diagnoses 1 to 7 months after test). More than three-fourths of patients with long COVID met our long COVID definition within 4 months of their positive COVID-19 test.

We found long COVID frequency differences based on race and ethnicity. These differences may be related to socioeconomic status, which is directly correlated with the presence of comorbidities [22-24]. The long COVID cohort was 8 years older with more comorbidities (2-year Elixhauser score 7.97 in the patients with long COVID vs 4.21 in the patients with non–long COVID). In our cohort, the men were significantly older than the women on average, 60.29 years (95% CI 60.24-60.35) versus 47.85 years (95% CI 47.73-47.97), respectively. We found that long COVID frequency was increased in patients who were more severely ill before infection and patients who had a more severe bout of COVID-19 as judged by their minimum oxygen saturation.

We found 143 upregulated diagnostic groups, with odds ratios as high as 23. We also found 17 upregulated medical specialty groupings containing between 3 and 21 signs, symptoms, or diagnoses. This provides strong evidence for a broad definition of long COVID.

Carfi et al [2] found that the most common long-term symptoms were fatigue, dyspnea, joint pain, and chest pain. Each except joint pain is represented in our long COVID definition. However, joint pain may be related to findings in our definition such as difficulty walking and an overall decrease in mobility. COVID-19 is known to cause lung abnormalities, especially in cases with pneumonia [25]. We found that the likelihood of developing pneumonia after COVID-19 infection is significantly upregulated, potentially interconnected with the numerous findings in our pulmonary long COVID definition. Autopsy evaluation of COVID-19 victims’ lung tissue demonstrated diffuse alveolar damage with perivascular T-cell infiltration and severe endothelial injury [26]. Patients with long COVID have been found to have abnormal ^129^Xe magnetic resonance imaging gas exchange and computed tomography vascular density measurements, which we postulate could be related to the pulmonary fibrosis (J84.10) or emphysema (J43.9) diagnoses identified in our definition [27].

Our definition shows that the long-term effects of COVID-19 are associated with damage to numerous body systems including the kidneys, heart, eyes, and nervous system. Our results are corroborated by other studies. Cognitive dysfunction (brain fog) is often associated with long COVID and can be difficult to diagnose and treat [5]. COVID-19 infection is far more likely to cause cardiac complications than vaccination [28]. The gastrointestinal codes we observed reflect previous literature [29] and may relate to reported alterations to the gastrointestinal tract after COVID-19 [3,4]. Finally, previous studies have noted that COVID-19 can alter ocular physiology, supporting our ophthalmology-related findings [30].

Patients with more severe cases of COVID-19, as manifested by low oxygen saturations, should be watched carefully for the development of long COVID as they are significantly more likely to develop long COVID. Sicker patients with higher 2-year Elixhauser scores were significantly more likely to develop long COVID. Patients with multiple comorbidities should be made aware of this risk and participate in active surveillance for the development of signs and symptoms of long COVID.

The American Medical Association notes there are 3 categories of patients with long COVID: those who do not recover completely and have ongoing symptoms, those with symptoms related to chronic hospitalization, and those who develop new symptoms after recovery [31]. In our study, we did not differentiate by these subtypes and instead leave that to future research. It is possible that some of these signs and symptoms may have occurred during the first month and may be the persistent subtype. It is possible that some of the upregulated codes may be found with other serious illnesses, though only 9.1% (33,314/367,148) of our cohort had severe COVID-19 based on oxygen saturation <94%. We are not able to distinguish conditions that represent an acceleration of pre-existing disease from those that represent de novo COVID-19–related conditions. For example, is the increased incidence of nonsore throat elevation myocardial infarction (I21.4) related to the general stress of acute illness impacting pre-existing coronary artery disease or to an underlying de novo long COVID–related condition? A better understanding will require additional research. In any event, whether causal or associative, de novo disease or exacerbation of chronic disease and new or persistent clinical problems require assessment, treatment, and monitoring.

Limitations include that the cohort study population is 84% men, reflective of the overall patient population of VA which is between 87% and 95% men (depending upon data source and whether gender has been self-reported) [32,33]. Additionally, the male veteran population who use the VA health care system is older than the population of female veterans who use the VA. Our study did not include home testing for COVID-19 that went unreported to the VA health care system. Patients who tested positive during the omicron dominant time period were slightly more likely to develop long COVID when compared to the earlier strains (66,643/87,522, 76% vs 201,677/279,626, 72%; *P*<.001). The reality of emerging viral variants emphasizes the need for a well-defined and well-maintained definition of long COVID over time and with variant-specific derivation. This study was not powered to show the independence of the individual risk factors for long COVID.

We hope that our empirically defined long COVID definition will lead to more consistent identification of long COVID and its medical specialty subtypes and support of a variety of COVID-19–related initiatives. Our definition is actionable as individuals who have multiple comorbidities and more severe bouts of COVID-19 should be followed more closely for the development of long COVID signs or symptoms. Our definition can also inform screening questions for high-risk patients. For example, helping clinicians identify patients with enhanced long COVID risk who may benefit from monitoring programs or patients with previously undiagnosed long COVID for whom it may be appropriate to create a referral to a long COVID clinic. We also anticipate that our long COVID definition will support the standardization of future subspecialty-specific long COVID research.

Future research should look at health outcomes for each long COVID-19 medical specialty subtype to identify those at greatest risk of developing severe morbidity. Predictive analytics should be used to help refer these individuals earlier to monitoring and treatment programs.

As of December 17, 2023, there have been over 772 million confirmed cases of COVID-19 worldwide [34]. Case counts are ever-increasing. As Levine [18] notes, immediately useful long COVID definitions are needed as are ultimately more fully inclusive definitions. We offer our long COVID definition as a public health contribution to our pandemic response.

## Appendix

Multimedia Appendix 1 Long COVID definition results by International Classification of Diseases, 10th Revision, Clinical Modification functional group description and medical specialty.

Multimedia Appendix 2 Combined odds ratios calculated for each medical specialty category.

Multimedia Appendix 3 Additional information about specific codes.

Multimedia Appendix 4 Data used to create these figures.

## Abbreviations

HIPAA: Health Insurance Portability and Accountability Act
ICD-10-CM: International Classification of Diseases, 10th Revision, Clinical Modification
VA: Veterans Affairs

## Data Availability

All data generated or analyzed during this study are either protected patient data that require a Veterans Affairs research appointment and institutional review board protocol to access or summarized and included in this published paper (and its supplementary information files).
