# Supplementary material for: Defining the Subtypes of Long COVID and Risk Factors for Prolonged Disease: Population-Based Case-Crossover Study
Source: JMIR Public Health Surveill. 2024 Apr 30;10:e49841. doi: 10.2196/49841 (PMC11094603; doi:10.2196/49841)
Supplement: Multimedia Appendix 1 [file publichealth_v10i1e49841_app1.docx]

**Table S4.** Long COVID definition results by ICD-10-CM functional group description and medical specialty. ICD-10-CM functional groups were created using multiple ICD-10-CM codes. Combined odds ratios were calculated for each medical specialty category in Table S2 in Multimedia Appendix 2. For additional information about specific codes, see Table S3 in Multimedia Appendix 3. CIs can be found in Table S4, Multimedia Appendix 4.

| Functional Group Description | Medical Specialty | ICD-10-CM Codes | Novel Count | Odds Ratio |
| --- | --- | --- | --- | --- |
| Hypotension | Cardiology | I95.1, I95.89, I95.9 | 7865 | 1.271538 |
| Heart Failure Diastolic Dysfunction | Cardiology | I11.0 | 4594 | 1.26876 |
| Cardiac Arrhythmia | Cardiology | I47.1, I48.0, I48.19, I48.20, I48.21, I48.3, I48.91, I48.92, I49.3, R00.0, R00.2 | 35648 | 1.223517 |
| Cardiovascular Disease | Cardiology | I21.A1, I24.8, I25.10, I25.2, I25.5, I42.8, I42.9, I50.20, I50.22, I50.23, I50.30, I50.32, I50.33, I50.42, I50.9, I71.40 | 45157 | 1.206416 |
| Valve Stenosis/Insufficiency | Cardiology | I27.82, I34.0, I35.0 | 3916 | 1.171221 |
| Peripheral Vascular Disease | Cardiology | I73.9 | 4862 | 1.105507 |
| Hyperlipidemia | Cardiology | E78.2, E78.5 | 54913 | 1.070793 |
| Hypertension | Cardiology | I10 | 39677 | 1.046517 |
| Gingival Recession | Dentistry | K06.010, K06.020 | 2497 | 1.256852 |
| Gingivitis | Dentistry | K03.0, K05.00, K05.10, K05.321, K05.322, K06.1 | 7818 | 1.235098 |
| Dental Caries | Dentistry | K02.3, K02.51, K02.52, K02.62, K02.7 | 19887 | 1.154138 |
| Pressue ulcer | Dermatology | L89.152, L89.153, L89.159 | 1639 | 2.3395 |
| Nonscarring Hair Loss | Dermatology | L65.9 | 441 | 1.853409 |
| Melanocytic Nevi | Dermatology | D22.9 | 1886 | 1.195428 |
| Nail Dystrophy | Dermatology | L60.3 | 5209 | 1.152928 |
| Xerosis Cutis | Dermatology | L85.3 | 4048 | 1.133044 |
| Fatigue | Endocrinology | G93.3 | 542 | 10.18039 |
| Malnutrition | Endocrinology | E43, E44.0, E46, E63.9, R63.8, R64 | 6372 | 1.856287 |
| Adult Failure to Thrive | Endocrinology | R62.7 | 1827 | 1.578271 |
| Phosphorus Metabolism Disorder | Endocrinology | E83.39 | 1306 | 1.297077 |
| Abnormal Weight Loss | Endocrinology | R63.4 | 4439 | 1.16755 |
| Osteoporosis | Endocrinology | M81.0 | 1442 | 1.120668 |
| Prediabetes | Endocrinology | R73.03 | 6402 | 1.105807 |
| Type 2 Diabetes Mellitus | Endocrinology | E11.22, E11.3291, E11.36, E11.40, E11.42, E11.51, E11.649, E11.65, E11.9 | 49454 | 1.104288 |
| Hypothyroidism | Endocrinology | E03.9 | 7059 | 1.050629 |
| Obesity | Endocrinology | E66.9 | 19041 | 1.038481 |
| Ileus | Gastroenterology | K56.7 | 693 | 1.585583 |
| Enterocolitis | Gastroenterology | A04.72 | 760 | 1.568205 |
| Elevation of Liver Transaminase Levels | Gastroenterology | R74.01 | 2117 | 1.515881 |
| Polyp of Stomach and Duodenum | Gastroenterology | K31.7 | 718 | 1.339522 |
| Ascites | Gastroenterology | R18.8 | 795 | 1.332139 |
| Esophagitis | Gastroenterology | K08.499, K20.80, K20.90, K22.89 | 3013 | 1.317627 |
| Portal Hypertension | Gastroenterology | K76.6 | 671 | 1.291358 |
| Dysphagia | Gastroenterology | R13.10, R13.11, R13.12, R13.13 | 13011 | 1.274248 |
| Constipation | Gastroenterology | K59.00, K59.03, K59.09 | 13376 | 1.224596 |
| Diseases of Stomach and Duodenum | Gastroenterology | K31.89 | 1394 | 1.193473 |
| Gastritis | Gastroenterology | K29.50, K29.70 | 2963 | 1.191019 |
| Gastrointestinal Hemorrhage | Gastroenterology | K92.2 | 2320 | 1.186902 |
| Diverticulosis | Gastroenterology | K57.30 | 5522 | 1.168767 |
| Melena | Gastroenterology | K92.1 | 2334 | 1.143609 |
| Cirrhosis of Liver | Gastroenterology | K74.60 | 1683 | 1.13734 |
| Gastro-Esophageal Reflux Disease | Gastroenterology | K21.00, K21.9 | 31020 | 1.086091 |
| Fatty Liver | Gastroenterology | K76.0 | 4912 | 1.081379 |
| Homelessness | General Internal Medicine | Z59.00, Z59.01, Z59.02, Z59.811, Z59.812, Z59.819, Z59.89 | 11511 | 1.456793 |
| Vitamin Deficiency | General Internal Medicine | E53.8, E55.9 | 21619 | 1.105953 |
| Sleep Disorders | General Internal Medicine | G47.00, G47.33 | 41147 | 1.090759 |
| Pulmonary Embolism without Acute Cor Pulmonale | Hematology | I26.99 | 2670 | 1.804971 |
| MGUS | Hematology | E88.09, R77.8 | 1273 | 1.542297 |
| Acute Embolism and Thrombosis | Hematology | I82.401, I82.402, I82.409, I82.890 | 2510 | 1.42562 |
| Pancytopenia | Hematology | D61.818 | 939 | 1.291209 |
| Thrombocytopenia | Hematology | D69.59, D69.6 | 3876 | 1.260737 |
| Abnormal Coagulation Profile | Hematology | R79.1 | 826 | 1.250246 |
| Anemia | Hematology | D50.0, D50.9, D53.9, D62, D63.0, D63.1, D63.8, D64.89, D64.9 | 30750 | 1.242379 |
| Abnormal White Blood Cell Count | Hematology | D72.829 | 3528 | 1.167524 |
| Infectious Sequelae | Infectious Diseases | B94.8 | 606 | 23.42175 |
| Viral Pneumonia | Infectious Diseases | J12.89 | 1604 | 2.478623 |
| Influenza | Infectious Diseases | J09.X2 | 547 | 2.193782 |
| Thrush | Infectious Diseases | B37.0 | 537 | 1.844434 |
| Sepsis | Infectious Diseases | A41.89, A41.9, R65.20, R65.21 | 6816 | 1.608247 |
| Pseudomonas | Infectious Diseases | B96.5 | 682 | 1.595698 |
| Enterococcus | Infectious Diseases | B95.2 | 884 | 1.594505 |
| Bacterial Pneumonia | Infectious Diseases | J15.9 | 2862 | 1.54475 |
| MRSA | Infectious Diseases | B95.62, Z22.322 | 1316 | 1.488166 |
| Bacteremia | Infectious Diseases | R78.81 | 2139 | 1.377602 |
| Klebsiella | Infectious Diseases | B96.1 | 728 | 1.376463 |
| MSSA | Infectious Diseases | B95.61, B95.7 | 1015 | 1.341265 |
| Proteus | Infectious Diseases | B96.4 | 511 | 1.336283 |
| E. Coli | Infectious Diseases | B96.20 | 1077 | 1.319389 |
| Osteomyelitis | Infectious Diseases | M86.9 | 1121 | 1.224393 |
| Urinary Tract Infection | Infectious Diseases | N39.0, T83.511A | 8553 | 1.161612 |
| Drug Toxicity | Nephrology | T38.0X5A, T45.1X5A | 1073 | 1.686641 |
| Dehydration | Nephrology | E86.1 | 959 | 1.293327 |
| Acute Kidney Failure | Nephrology | N17.0, N17.8, N17.9 | 11392 | 1.290399 |
| Disorders of Fluid, Electrolyte and Acid-Base Balance | Nephrology | E83.42, E83.52, E87.0, E87.1, E87.2, E87.20, E87.3, E87.4, E87.5, E87.6, E87.70, E87.8 | 25885 | 1.276923 |
| Chronic Kidney Disease | Nephrology | I12.0, I12.9, I13.0, I13.2, N18.2, N18.30, N18.31, N18.32, N18.4, N18.6, N18.9, N28.89, Z99.2 | 35590 | 1.258035 |
| Edema | Nephrology | R60.0, R60.9 | 11683 | 1.116561 |
| Encephalopathy | Neurology | G92.8, G93.40, G93.41, G93.49 | 4476 | 1.705173 |
| Delirium | Neurology | F05 | 1414 | 1.639297 |
| Lower Back Pain | Neurology | M54.50 | 24694 | 1.578581 |
| Muscle Weakness | Neurology | M62.81 | 9362 | 1.277051 |
| Need for Assistance with Personal Care | Neurology | Z74.1 | 3287 | 1.276263 |
| Weakness | Neurology | R53.1, R54 | 15912 | 1.258534 |
| Cognitive Impairment | Neurology | R41.0, R41.81, R41.82, R41.841, R41.89, R41.9 | 10045 | 1.241561 |
| Wheelchair | Neurology | Z99.3 | 654 | 1.234073 |
| Seizure Disorder | Neurology | G40.909 | 1155 | 1.205124 |
| Dementia | Neurology | F01.50, F02.80, F03.90, G30.9, G31.84 | 10406 | 1.204819 |
| Falls | Neurology | R29.6 | 1879 | 1.199129 |
| Difficulty in Walking | Neurology | R26.2, R26.89, R26.9, Z74.09 | 22449 | 1.171356 |
| Falling | Neurology | Z91.81 | 3164 | 1.157633 |
| Tinnitus | Neurology | H93.13 | 8889 | 1.135359 |
| Cerebrovascular Disease | Neurology | I63.9, I69.351, I69.354 | 4200 | 1.119552 |
| Neuropathy | Neurology | G62.9, G89.18, G89.29, G89.3, G89.4 | 23681 | 1.118604 |
| Cervicalgia | Neurology | M54.2 | 13691 | 1.052616 |
| Malignant Neoplasm of Liver | Oncology | C78.7 | 420 | 1.704389 |
| Malignant Neoplasm of Lung | Oncology | C34.90 | 657 | 1.350535 |
| Malignant Neoplasm of Bone | Oncology | C79.51 | 622 | 1.314306 |
| Solitary Pulmonary Nodule | Oncology | R91.1 | 5557 | 1.116382 |
| Conjunctivitis | Ophthalmology | H10.45 | 2645 | 1.180325 |
| Retinopathy | Ophthalmology | H35.372, H35.373 | 2413 | 1.173309 |
| Dry Eye Syndrome | Ophthalmology | H04.123 | 19581 | 1.164934 |
| Presbyopia | Ophthalmology | H52.4 | 36752 | 1.154351 |
| Myopia | Ophthalmology | H52.13 | 8012 | 1.151422 |
| Vitreous Degeneration | Ophthalmology | H43.813 | 3085 | 1.142235 |
| Astigmatism | Ophthalmology | H52.203, H52.223 | 13737 | 1.140477 |
| Unspecified Disorder of Refraction | Ophthalmology | H52.7 | 7093 | 1.126198 |
| Open Angle with Borderline Findings | Ophthalmology | H40.013 | 6278 | 1.121644 |
| Cataracts | Ophthalmology | H25.012, H25.11, H25.12, H25.13, H25.811, H25.812, H25.813, H26.9 | 42904 | 1.119152 |
| Hypermetropia | Ophthalmology | H52.03 | 7317 | 1.110326 |
| Epistaxis | Otolaryngology | R04.0 | 1342 | 1.235389 |
| Dysphonia | Otolaryngology | R49.0 | 1368 | 1.182724 |
| Impacted Cerumen | Otolaryngology | H61.23 | 3780 | 1.121918 |
| Sensorineural Hearing Loss | Otolaryngology | H90.3, H90.A22 | 17093 | 1.1138 |
| Restlessness and Agitation | Psychiatry / Psychology | R45.1 | 893 | 1.279866 |
| Mental Disorder | Psychiatry / Psychology | F09, F32.A, F41.1, F41.9, F43.12 | 48256 | 1.135075 |
| Malaise | Psychiatry / Psychology | R53.81 | 2251 | 1.132321 |
| Problems Related to Psychosocial Circumstances | Psychiatry / Psychology | Z65.8, Z65.9 | 23892 | 1.102123 |
| Acute Respiratory Distress Syndrome | Pulmonary | J80 | 688 | 5.299347 |
| Dependence on Ventilator | Pulmonary | Z99.11 | 427 | 3.146519 |
| Chronic Cough | Pulmonary | R05.3 | 2113 | 2.026076 |
| Respiratory Failure | Pulmonary | J96.00, J96.01, J96.02, J96.10, J96.11, J96.20, J96.21, J96.22, J96.90, J96.91 | 14882 | 1.997749 |
| Dependence on Supplemental Oxygen | Pulmonary | R09.02, Z99.81 | 7792 | 1.715279 |
| Interstitial Pulmonary Disease | Pulmonary | J84.9 | 685 | 1.536233 |
| Pulmonary Fibrosis | Pulmonary | J84.10 | 1232 | 1.527598 |
| Pleural Effusion | Pulmonary | J90, J91.8 | 2990 | 1.4413 |
| Abnormalities of Breathing | Pulmonary | R06.89 | 1107 | 1.338466 |
| Pneumonia | Pulmonary | J16.8, J17, J18.8, J18.9, J69.0 | 8181 | 1.315208 |
| Acute Pulmonary Edema | Pulmonary | J81.0 | 624 | 1.295683 |
| Pulmonary Hypertension | Pulmonary | I27.20 | 1782 | 1.282603 |
| Respiratory Disorders | Pulmonary | J98.4, J98.8, J98.9 | 3711 | 1.281901 |
| Emphysema | Pulmonary | J43.9 | 1700 | 1.229811 |
| Dyspnea | Pulmonary | R06.00, R06.02, R06.03, R06.09 | 27926 | 1.206525 |
| Atelectasis | Pulmonary | J98.11 | 1766 | 1.206262 |
| Diaphragmatic Hernia | Pulmonary | K44.9 | 2952 | 1.169074 |
| Chronic Obstructive Pulmonary Disease | Pulmonary | J44.0, J44.9 | 12639 | 1.131736 |
| Asthma | Pulmonary | J45.909 | 4126 | 1.093696 |
| Osteoarthritis | Rheumatology | M17.0 | 4546 | 1.090886 |
| Gout | Rheumatology | M10.9 | 5997 | 1.057874 |
| Uropathy | Urology | N13.9 | 597 | 1.256139 |
| Retention of Urine | Urology | R33.8, R33.9 | 6269 | 1.251816 |
| Obstructive and Reflux Uropathy | Urology | N13.8 | 1385 | 1.204741 |
| Urinary Incontinence | Urology | N31.9, R32 | 3843 | 1.153016 |
| Benign Prostatic Hyperplasia | Urology | N40.0, N40.1 | 24210 | 1.128437 |
| Frequency of Micturition | Urology | R35.0 | 3032 | 1.106673 |
